# A case report of myxoma within the right submandibular muscle and a literature review

**DOI:** 10.3389/fsurg.2025.1733176

**Published:** 2026-01-22

**Authors:** Dingyu Tian, Xiao Liang, Juntao Ma, Ye Li, Yuliang Zhang, Rui Zhang

**Affiliations:** 1 Department of Stomatology, First Affiliated Hospital of Dalian Medical University, Dalian, Liaoning, China; 2 Department of Radiology, First Affiliated Hospital of Dalian Medical University, Dalian, Liaoning, China; 3 Department of Pathology, First Affiliated Hospital of Dalian Medical University, Dalian, Liaoning, China

**Keywords:** head and neck surgery, intramuscular myxoma, mylohyoid muscle, soft tissue tumor, submandibular region

## Abstract

**Objective:**

To investigate the etiology, clinical presentation, management, and prognosis of intramuscular myxoma occurring in the submandibular region.

**Methods:**

A case of intramuscular myxoma originating from the mylohyoid muscle was analyzed. The patient's clinical history, imaging features, surgical findings, and pathological results were reviewed. Relevant characteristics were summarized in the context of previously published literature.

**Results:**

The patient presented with a painless mass in the submandibular area. Computed tomography revealed a cystic lesion, and postoperative histopathological examination confirmed the diagnosis of intramuscular myxoma. The patient recovered well following surgical excision.

**Conclusion:**

Intramuscular myxoma is a rare benign soft-tissue tumor, with an estimated incidence of approximately 0.10–0.13 per 100,000 individuals. Lesions arising in the submandibular muscles are exceptionally uncommon and may be misdiagnosed as sublingual gland cysts. Due to the nonspecific clinical manifestations and potential for misdiagnosis on imaging, histopathological evaluation remains the definitive diagnostic method. Complete surgical excision is the treatment of choice and is generally associated with a favorable prognosis.

## 1 Introduction

Intramuscular myxoma (IM) is a benign mesenchymal tumor that arises within skeletal muscle, characterized by sparsely cellular, spindle-shaped nuclei embedded in abundant myxoid stroma with relatively few blood vessels (1). It most commonly occurs in adults aged 40–70 years, with a higher prevalence in females, and predominantly affects the striated muscles of the thighs (2). In contrast, IM in the submandibular region is extremely rare. Its anatomical location can closely mimic an extraoral variant of a sublingual gland cyst, and the absence of specific immunohistochemical markers further complicates differentiation. Compared with salivary gland tumors, IM is far less common, increasing the likelihood of clinical misdiagnosis.

This report presents a case of IM originating in the submandibular region. The patient's clinical presentation, imaging findings, and pathological characteristics are comprehensively analyzed to enhance diagnostic awareness among clinicians, pathologists, and head and neck radiologists. Accurate identification of this entity may help prevent unnecessary interventions and reduce the potential physical, psychological, and financial burdens associated with misdiagnosis.

## 2 Materials and methods

### 2.1 Case presentation

A 60-year-old man was admitted to the hospital with a complaint of a palpable mass in the right side of the neck for over one month. He stated that the mass was discovered incidentally and was approximately the size of a walnut at the time of detection. He reported no associated pain, dysphagia, or other discomfort. He self-administered antibiotics of unknown type and dosage without noticeable improvement and therefore did not seek medical attention initially.

On the day of admission, the patient reported: “I accidentally noticed a walnut-sized mass on the right side of my neck about one month ago. As it was painless, I did not pay much attention to it at the time. Recently, at the urging of my family, I sought medical consultation at the hospital”. The patient had been previously healthy, with no history of chronic illnesses, neck surgery, trauma, or local radiotherapy. He denied any history of infectious diseases, including hepatitis, syphilis, or HIV infection, and reported no drug, food, or contact allergies. His immunizations were consistent with the local vaccination program, although specific details were unavailable.

### 2.2 Preliminary diagnosis and treatment

A palpable mass was identified inferior to the right mandibular angle, measuring approximately 4.0 × 3.0 cm. The lesion exhibited clear margins, a firm but mobile texture, and no tenderness upon palpation. No positional changes were noted, and the overlying skin appeared normal. Bimanual examination revealed symmetrically sized submandibular glands with a soft texture. Clear saliva could be expressed from the submandibular duct opening, and no redness or swelling was observed at the duct orifice. No cervical or submental lymphadenopathy was detected. The remainder of the clinical examination was unremarkable. As MRI was not performed due to objective limitations, contrast-enhanced CT was obtained. Contrast-enhanced axial CT demonstrated a well-defined low-attenuation cystic mass located in the right submandibular space, positioned medially to the mylohyoid muscle (mm. mylohyoideus) and deep to the submandibular gland. The lesion measured approximately 5.3 × 2.0 × 7.0 cm. Ultrasound-guided fine-needle aspiration (FNA) was performed. A small amount of clear, slightly viscous mucoid material was obtained and submitted for cytological examination, which suggested a neoplastic lesion but was inconclusive due to insufficient sample volume. A second FNA yielded a jelly-like specimen. Histopathological analysis revealed fibromyxoid tissue, raising suspicion for a myxoid neoplasm.

Based on the patient's physical examination upon admission, puncture findings, and imaging results, the preliminary diagnosis was a mass in the right submandibular region. A “right submandibular mass resection” was scheduled. During the surgery, an approximately 8 cm incision was made 2 cm below the lower margin of the right mandible, extending forward from the mandibular angle. A grayish-yellow, jelly-like mass was observed. The mass was located superior to and closely adjacent to the submandibular gland, which appeared normal in texture. Due to the incomplete capsule of the mass, it was adherent to the surrounding mylohyoid muscle. The mass was completely excised and submitted for intraoperative frozen section analysis. The frozen section indicated a mucus-rich benign lesion in the right submandibular region. The surgery proceeded smoothly. After the operation, anti-inflammatory, anti-swelling, and hemostatic treatments were administered along with local cryotherapy. Histopathological evaluation revealed a poorly defined border in most areas, with infiltration into skeletal muscle arranged in a checkerboard-like pattern. Tumor cellularity was low, and the spindle-shaped and stellate cells exhibited no significant atypia. Myxoid stroma was prominent, with focal microcystic changes. The diagnosis of cellular intramuscular myxoma was initially considered, and close follow-up was recommended. Three lymph nodes showed reactive hyperplasia.

Immunohistochemical results: Bcl-2 (−), CD34 (+), ERG (vascular +), Ki-67 (hotspot +10%), P63 (−), S-100 (−), SMA (−), SOX-10 (−).

A palpable mass was detected inferior to the right mandibular angle, measuring approximately 4.0 × 3.0 cm. The lesion had well-defined margins, a firm yet mobile consistency, and was non-tender on palpation. No positional changes were observed, and the overlying skin appeared normal. Bimanual examination revealed symmetrically sized submandibular glands with a soft consistency. Clear saliva could be expressed from the submandibular duct orifice, which showed no signs of redness or swelling. No cervical or submental lymphadenopathy was noted. The remainder of the physical examination was unremarkable. Based on these clinical features, a benign tumorous lesion was considered the preliminary diagnosis. Acute infectious conditions, such as lymphadenitis, were excluded because these typically present with redness, swelling, pain, and respond to anti-infective therapy, none of which were consistent with this case. Conversely, highly malignant tumors, such as myxoid sarcoma, usually manifest as rapidly enlarging masses with ill-defined borders and associated pain, and were therefore also deemed unlikely. To further evaluate the lesion and delineate its boundaries, the patient underwent contrast-enhanced computed tomography (CT) of the salivary glands on the third day after admission. CT imaging revealed an oval, well-circumscribed, low-attenuation mass in the right submandibular region, following the course of the mylohyoid muscle.

**Figure 1:**
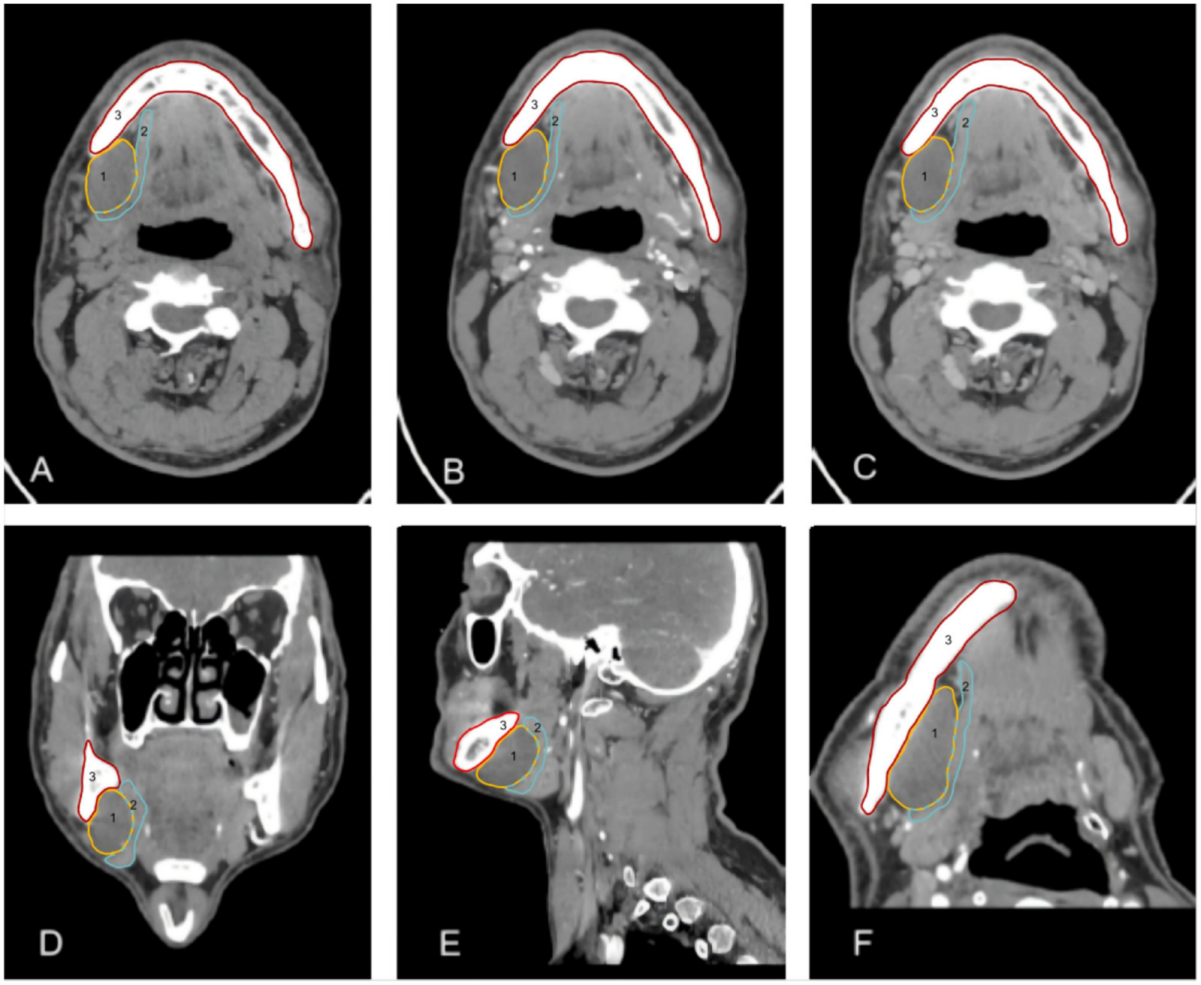
**(A–C)** represent the non-contrast, arterial-phase, and venous-phase CT scans of the salivary glands, respectively. **(D)** is a coronal reconstruction of the arterial phase, and **(E)** is an additional arterial-phase sectional image. **(F)** is a curved planar reconstruction image along the course of the right mylohyoid muscle in the arterial phase. The red area indicates the mandible, the yellow area denotes the lesion site, and the blue area represents the mylohyoid muscle.

**Figure 2:**
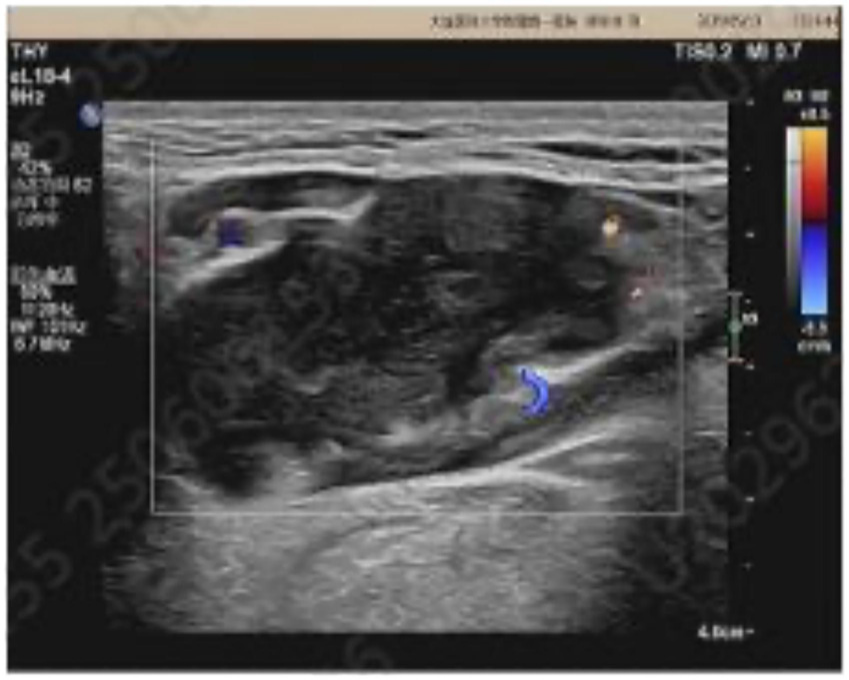
A mixed-echo lesion was identified in the right submandibular region, with well-defined borders and a relatively regular shape. Blood flow signals were observed within the solid component.

The lesion measured approximately 5.3 × 2.0 × 7.0 cm. On non-contrast CT, it appeared as a homogeneously low-density mass with an average attenuation of approximately 18.3 HU, showing no significant enhancement on contrast-enhanced scans (CT values: 21.2 HU in the arterial phase and 20.5 HU in the venous phase). The interface between the lesion and the medial aspect of the mylohyoid muscle was indistinct. Mild compression and thinning of the mylohyoid muscle were observed, along with displacement of the right submandibular gland and masseter muscle. No evidence of bone erosion or cortical disruption was detected in the right mandibular body. A small lymph node, measuring approximately 0.4 cm in short-axis diameter, was visualized in the right level Ib region (Figure 1). Ultrasound examination was performed on the seventh day after admission. The right salivary gland exhibited homogeneous echotexture and normal volume. A mixed-echo lesion was identified in the right submandibular region, measuring approximately 43 × 24 mm, with well-defined borders and a relatively regular shape. Blood flow signals were observed within the solid component (Figure 2). Ultrasound-guided fine-needle aspiration (FNA) was performed concurrently. A small amount of clear, slightly viscous mucoid material was obtained and submitted for cytological examination, which suggested a neoplastic lesion but was inconclusive due to insufficient sample volume. A second FNA yielded a jelly-like specimen. Histopathological analysis revealed fibromyxoid tissue, raising suspicion for a myxoid neoplasm.

Based on the above auxiliary examinations, lymphadenopathy (which typically presents with a target-ring appearance and a visible hilum) and cutaneous-derived neoplasms (which are usually superficial and adherent to subcutaneous tissue) were ruled out. Given the indistinct interface between the lesion and the medial aspect of the mylohyoid muscle on contrast-enhanced CT, the mass was suspected to be a mucus-rich benign tumor of muscular origin.

**Figure 3:**
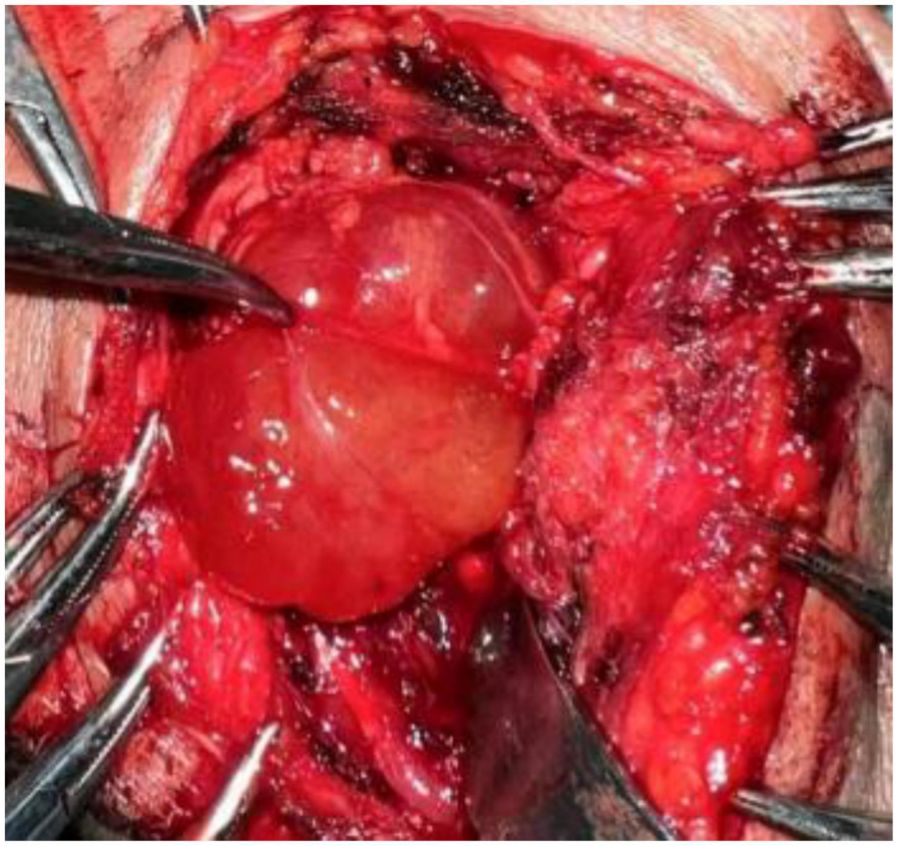
A grayish-yellow, jelly-like mass was identified intraoperatively.

**Figure 4:**
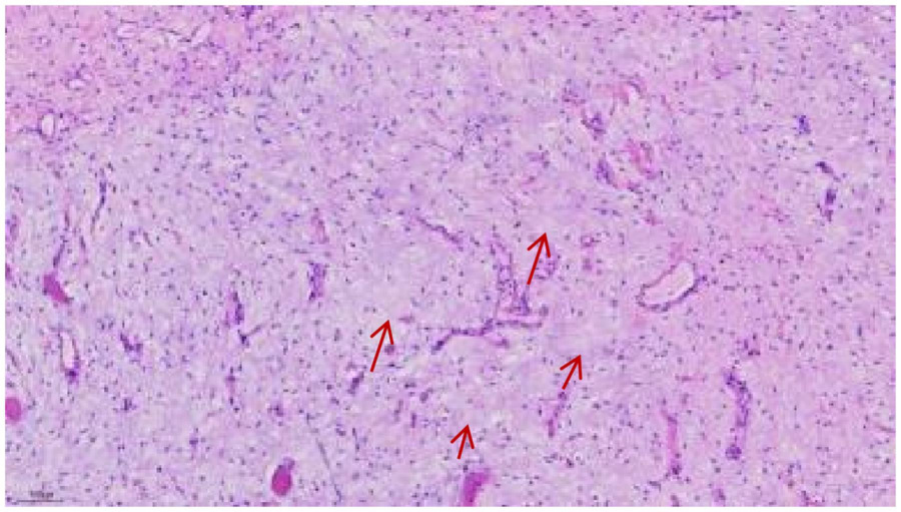
Scattered tumor cells were present within abundant myxoid stroma, which exhibited focal microcystic changes. The arrow indicates the myxoid stroma.

After comprehensive evaluation, the medical team recommended surgical excision. The patient's family was informed of the procedure and potential risks in detail, and informed consent was obtained. Three days later, the patient underwent radical resection of the right submandibular mass under general anesthesia. During surgery, an approximately 8 cm incision was made 2 cm below the inferior margin of the right mandible, extending anteriorly from the mandibular angle. A grayish-yellow, jelly-like mass was identified (Figure 3). The mass was situated superior to and closely adjacent to the submandibular gland, which appeared normal in texture. Due to the incomplete capsule, the lesion was adherent to the surrounding mylohyoid muscle. The mass was completely excised and submitted for intraoperative frozen section analysis, which indicated a mucus-rich benign lesion. The procedure was completed smoothly. Postoperatively, the patient received anti-inflammatory, anti-edema, and hemostatic treatments, along with local cryotherapy. Histopathological evaluation on postoperative day 7 revealed that most borders of the lesion were ill-defined, with infiltration into skeletal muscle in a checkerboard-like pattern. Scattered tumor cells were present within abundant myxoid stroma, which exhibited focal microcystic changes. Tumor cells were sparsely distributed, spindle- or stellate-shaped, without significant atypia. The nuclei were small, hyperchromatic, round or oval, and the cytoplasm was scant, lightly stained, with indistinct borders. Immunohistochemical analysis showed: Bcl-2 (−), CD34 (+), ERG (vascular +), Ki-67 (hotspot ∼10%), P63 (−), S-100 (−), SMA (−), SOX-10 (−) (Figure 4).

## 3 Results

Based on the patient's medical history, clinical examination, admission CT findings, floor-of-mouth puncture results, and postoperative pathological evaluation, the final diagnosis was intramuscular myxoma in the right submandibular region. After surgery, the patient exhibited no significant swelling or other discomfort in the operative area. The drainage tube was removed 3 days postoperatively, and the patient was discharged home. The patient's family was instructed to return for follow-up once a week initially, and subsequently at three months, six months, and one year after the operation.

Based on the aforementioned findings, a final diagnosis of cellular intramuscular myxoma was established. The patient reported that the surgical incision had healed well, with normal eating and speech resumed, and no discomfort. Follow-up visits were scheduled at 1, 3, and 6 months postoperatively. At the 1-month follow-up, the patient was in good mental condition and reported no pain or numbness at the surgical site. At 3 months postoperatively, the patient noted gradual fading of the cervical surgical scar, with no limitations in daily diet or activities. At 6 months postoperatively, the patient remained completely asymptomatic and had fully resumed normal work and daily activities. The surgical scar appeared as a white linear stripe without any hypertrophic changes. Ultrasonography demonstrated clear tissue structures in the surgical area, with no evidence of local recurrence.

## 4 Discussion

### 4.1 Etiology and pathogenesis

Intramuscular myxoma (IM) is a subtype of myxoma, sharing an identical pathogenesis. Most researchers believe that it originates from primitive embryonic stroma or fibroblasts. Post-traumatic secondary infection and chronic irritation have also been suggested as potential predisposing factors (3). This tumor predominantly arises in muscle-rich regions, such as the buttocks and lower extremities. In the head and neck region, IM most commonly occurs in the jawbones, but it may also develop in the buccal region and other sites. It typically presents as a solitary, painless mass; however, when the tumor enlarges sufficiently to compress adjacent nerves or blood vessels, corresponding clinical symptoms may occur. The majority of soft tissue myxomas in the head and neck region are of muscular origin (4, 5).

### 4.2 Imaging characteristics

#### 4.2.1 Ultrasound

The ultrasonic features of intramuscular myxoma (IM) typically manifest as solitary, oval, well-defined intramuscular lesions, with their long axis oriented parallel to the direction of the muscle fiber bundles. Triangular or ring-shaped enhanced echoes observed at both ends of the tumor are characteristic, often referred to as the “bright cap sign” or “bright edge sign”. The underlying mechanism involves infiltration of the mucinous matrix into surrounding adipose tissue, which compresses adjacent muscle fibers, leading to muscular atrophy and edema. In the present case, the “bright cap sign” was not prominent, likely due to the tumor's slow growth and the short duration of the disease.

#### 4.2.2 Computed tomography (CT)

On CT, IM usually appears as a cystic, low-density lesion.

#### 4.2.3 Magnetic resonance imaging (MRI)

MRI is the preferred imaging modality for diagnosing IM. Typical MRI findings include a well-defined, oval soft tissue mass that is generally hypointense on T1-weighted images (T1WI) and markedly hyperintense on T2-weighted and T2 fat-saturated sequences, without diffusion restriction on diffusion-weighted imaging (DWI), resembling a cystic lesion. This imaging appearance reflects the abundant myxoid stroma within the tumor (6). Despite these characteristic features, differentiation from other entities is necessary. For example, a defect in the mylohyoid muscle can allow the submandibular gland to herniate into adjacent spaces, creating a pseudo-mass on imaging. Such anatomical variations mimic true pathological lesions but lack intrinsic muscular characteristics (7). Myxoid liposarcoma typically presents with more heterogeneous signals, pronounced enhancement, and may contain fat components (hyperintense on T1WI, with signal suppression on fat-saturated sequences). Schwannomas may also exhibit high T2 signal intensity but are usually associated with nerve trunks and may demonstrate a “target sign”, characterized by low central signal in fibrous tissue and high peripheral signal in the myxoid matrix (6).

### 4.3 Pathological characteristics

Intramuscular myxoma (IM) is classified pathologically into classic myxoma, cell-rich myxoma, and syndrome-associated myxoma. Grossly, the tumors are typically light gray or light yellow, oval-shaped, and exhibit a shiny, mucus-like appearance, with either absent or incomplete capsules. Under light microscopy, the tumor is composed of sparsely distributed small stellate or spindle-shaped cells embedded within abundant myxoid stroma. The lesion may demonstrate local invasiveness. Electron microscopy reveals that the tumor cells are fully fibroblastic, and the stroma consists of abundant mucinous substance, with a network of reticular and collagen fibers interspersed within it (8). Immunohistochemically, Vimentin and CD34 are generally positive, while S-100 is typically negative (9).

Hematoxylin and eosin (H&E) staining of myxomas demonstrates a characteristic myxoid stroma, appearing as a homogeneous pale blue or pale pink matrix, within which a small number of morphologically bland spindle or stellate cells are scattered. These cells exhibit delicate nuclear chromatin, inconspicuous nucleoli, and no significant atypia or mitotic activity. These histological features clearly distinguish IM from malignant tumors such as myxofibrosarcoma, which typically contain highly atypical cells and numerous mitoses. Thus, H&E staining serves as an initial and essential tool for differentiating myxomas from other myxoid lesions, providing a key basis for diagnosis by clearly demonstrating their characteristic histological and cytological morphology (10).

### 4.4 Differential diagnosis

#### 4.4.1 Sublingual gland cyst

Sublingual gland cysts are classified into three types: simplex, plunging, and mixed, with the plunging type most prone to misdiagnosis as a cervical mass. Clinically, this type presents as a painless, soft mass in the submandibular region. On computed tomography (CT), it typically appears as a unilocular cystic lesion with a very thin wall and a uniformly hypodense cystic cavity. Previous studies have reported that, because the lesion of a plunging sublingual gland cyst extends from the anterior portion of the sublingual space, a characteristic narrow “tail-like” structure can often be observed on imaging, with the main lesion predominantly located in the submandibular space. However, this feature serves only as a valuable diagnostic clue and is not a mandatory criterion for definitive diagnosis. Given the challenges in differentiating sublingual gland cysts from other maxillofacial cystic lesions on imaging alone, clinicians often combine auxiliary diagnostic methods, such as fine-needle aspiration biopsy and salivary amylase assays, to improve diagnostic sensitivity and specificity (11).

#### 4.4.2 Sublingual dermoid and epidermoid cysts

Both dermoid and epidermoid cysts are congenital dysembryoplastic lesions that typically present as slowly enlarging, well-circumscribed, round or oval masses. On CT, the density of the cyst contents varies according to tissue composition, appearing as homogeneous or heterogeneous density shadows. Dermoid cysts, in particular, may contain skin appendages or other components within the cyst cavity, resulting in characteristic fat-density areas. Additionally, identification of epithelial cells in aspirated material via fine-needle aspiration cytology (FNAC) provides strong evidence for diagnosis (12).

#### 4.4.3 Schwannoma

Schwannomas are most often located along the course of peripheral nerves and may present with pain or numbness in the lesion area; palpation can induce radiating pain along the nerve trajectory. On CT, they appear as well-demarcated, round masses with homogeneous density, occasionally containing areas of cystic degeneration. Contrast-enhanced scans often demonstrate characteristic rim enhancement of the tumor capsule (13).

#### 4.4.4 Myxoid sarcoma

Myxoid sarcoma is a rare type of soft tissue sarcoma, characterized by rapid tumor growth, adhesion to surrounding tissues, and poor mobility. As the tumor enlarges, it can compress adjacent tissues, nerves, or blood vessels, leading to corresponding symptoms such as dysfunction and pain. On CT scans, it manifests as a heterogeneous density mass with interspersed solid components and cystic degeneration areas, ill-defined borders, and frequent invasion of adjacent muscles, fascia, and even bones. Marked enhancement of the solid components is observed on contrast-enhanced scans (14, 15).

This study has two main limitations. First, although magnetic resonance imaging (MRI) is widely recognized as the preferred modality for the differential diagnosis of deep soft tissue lesions in the maxillofacial region, only computed tomography (CT) was employed in this study due to objective constraints. Consequently, the accuracy in qualitatively differentiating lesions with similar densities was limited, and the diagnostic efficacy was inferior to that of studies utilizing combined multimodal imaging. Second, fine-needle aspiration cytology (FNAC) combined with salivary amylase assay is a key adjunctive method for differentiating ranula from intramuscular myxoma and enhancing diagnostic sensitivity. However, due to the high viscosity of the lesion contents, the sample volume obtained via FNAC was limited, resulting in insufficient cytological evidence to fully support the conclusions.

### 4.4 Treatment and prognosis

Intramuscular myxoma is a rare benign soft tissue tumor, with an estimated incidence of 0.10–0.13 cases per 100,000 population (9). Clinically, it typically presents as a painless mass, and pathological examination remains the gold standard for definitive diagnosis. Surgical resection is the treatment of choice. The overall postoperative recurrence rate is relatively low, approximately 3%–8%, with most recurrences occurring within the first two years after surgery. Therefore, close follow-up and dynamic imaging surveillance are recommended. In conclusion, accurate clinical diagnosis of intramuscular myxoma requires a combination of detailed imaging evaluation and pathological assessment. For clinical management, emphasis should be placed on complete tumor excision. Postoperatively, individualized follow-up and rehabilitation plans should be developed to monitor recurrence risk, minimize recurrence rates, and optimize patient outcomes (5, 16).

## Data Availability

The original contributions presented in the study are included in the article/Supplementary Material, further inquiries can be directed to the corresponding author.
